# Transvaginal Bilateral Sacrospinous Fixation after Second Recurrence of Vaginal Vault Prolapse: Efficacy and Impact on Quality of Life and Sexuality

**DOI:** 10.1155/2018/5727165

**Published:** 2018-02-28

**Authors:** Salvatore Giovanni Vitale, Antonio Simone Laganà, Marco Noventa, Pierluigi Giampaolino, Brunella Zizolfi, Salvatore Butticè, Valentina Lucia La Rosa, Giuseppe Gullo, Diego Rossetti

**Affiliations:** ^1^Unit of Gynecology and Obstetrics, Department of Human Pathology in Adulthood and Childhood “G. Barresi”, University of Messina, Via Consolare Valeria 1, 98125 Messina, Italy; ^2^Department of Obstetrics and Gynecology, “Filippo Del Ponte” Hospital, University of Insubria, Piazza Biroldi 1, 21100 Varese, Italy; ^3^Department of Woman and Child Health, University of Padua, Via Giustiniani 3, 35128 Padua, Italy; ^4^Department of Public Health, School of Medicine, University of Naples “Federico II”, Via Pansini 5, 80131 Naples, Italy; ^5^Department of Human Pathology, Unit of Urology, University of Messina, Via Consolare Valeria 1, 98125 Messina, Italy; ^6^Unit of Psychodiagnostics and Clinical Psychology, University of Catania, Via Santa Sofia 78, 95124 Catania, Italy; ^7^Unit of Gynecology and Obstetrics, Desenzano del Garda Hospital, Section of Gavardo, Via A. Gosa 74, Gavardo, 25085 Brescia, Italy

## Abstract

**Objective:**

Our aim was to study the efficacy of transvaginal bilateral sacrospinous fixation (TBSF) and its impact on quality of life (QoL) and sexual functions in women affected by second recurrences of vaginal vault prolapse (VVP).

**Materials and Methods:**

We performed a prospective observational study on 20 sexually active patients affected by second recurrence of VVP, previously treated with monolateral sacrospinous fixation. TBSF was performed in all the patients. They had been evaluated before the surgery and at 12-month follow-up through pelvic organ prolapse quantification (POP-Q) system, Short Form-36 (SF-36), and Pelvic Organ Prolapse/Urinary Incontinence Sexual Questionnaire (PISQ-12).

**Results:**

At 12-month follow-up, 18 out of 20 (90%) patients were cured of their recurrent VVP. No major intra- and postoperative complications occurred. We found a significant improvement in 4/5 POP-Q landmarks (excluding total vaginal length), SF-36, and PISQ-12 scores.

**Conclusion:**

According to our data analysis, TBSF appears to be safe, effective, and able to improve both QoL and sexual functions in patients affected by second recurrence of VVP after previous monolateral sacrospinous fixation.

## 1. Introduction

Second recurrence of vaginal vault prolapse (VVP) is defined as prolapse of the vaginal vault or upper vagina after two previous reconstructive surgeries; this occurs when the top of the vagina descends below a point that is 2 cm less than the total vaginal length above the plane of the hymen [1]. Risk factors for VPP may include age, increased body max index (BMI), chronic lung disease, preoperative pelvic-organ-prolapse (POP) stage 3 or higher, and impaired correction of anatomic defects at surgery [2, 3].

The two most accepted surgical techniques for primary VPP are laparoscopic sacrocolpopexy (LSC) and sacrospinous fixation (SF) [4]. No randomized controlled trials have been published comparing the efficacy of the two treatments; however, LSC seems to be correlated to a lower recurrence rate of VVP and less dyspareunia; SF is associated with a shorter operation time, lower costs, and an earlier return to daily activities [5].

In case of second recurrence VPP data are even more contrasting, and there is no consensus regarding the ideal surgical procedure [6]. On one hand, there is little information regarding long-term general quality of life (QoL) and sexual function in patients with primary VPP treated by LSC or SF [4, 7], and on the other there are no data available concerning the treatment of second recurrence. Sexual wellbeing is an important aspect of women's health; therefore, sexual disorders can decrease the QoL.

Pelvic floor weakness and organ prolapse are common physical conditions that negatively affect sexual functions and satisfaction [8–11]. Although some authors have reported that the surgical treatment of VVP improves sexual functions [12], its real impact has not been definitively confirmed [13].

As mentioned above, SF is one of the most common and effective surgical procedures for the treatment of VVP. It was first described in 1968 [14] and, initially, it was performed unilaterally on the sacrospinous ligament, following dissection of the pararectal space. Over time, in order to reduce dyspareunia and bowel symptoms due to the deviation of the vaginal axis [15], the bilateral technique (transvaginal bilateral sacrospinous fixation, TBSF) was gradually introduced to treat prolapse of the uterus, vaginal apex, and recurrent vaginal vault prolapse [16].

To the best of our knowledge there are no studies defining the best surgical technique for the treatment of second recurrence VVP, and as a result a standard procedure is still lacking. Therefore, the aim of this study was to evaluate the feasibility, safety, and efficacy of TBSF for the treatment of second recurrence VVP after previous monolateral SF and its impact on QoL and sexual functions.

## 2. Materials and Methods

### 2.1. Study Design

We carried out an observational prospective study on patients who were diagnosed with second recurrence of VVP after previous monolateral SF, referred to the Gynecology Department of Azienda Socio Sanitaria Territoriale- (ASST-) Garda (Italy) from January 2008 to December 2014.

An independent Institutional Review Board approved this prospective observational study. All of the women were given a written informed consent before entering the study, which was conducted in accordance with the Declaration of Helsinki. The study was not advertised and no remuneration was offered.

### 2.2. Inclusion and Exclusion Criteria

We included all newly diagnosed patients with symptomatic second recurrence of VVP (sensation of heaviness or pulling in the pelvis, tissue protruding from the vagina, discomfort during sex, urine leakage in case of cough, sneezing or exercise, and urine retention) after previous vaginal hysterectomy and correction of primary VPP with transvaginal monolateral SF.

All of the women included underwent TBSF and were reevaluated after 12 months of follow-up. Additional factors for the inclusion criteria were POP stage II or higher according to the POP-Q system [17], menopausal status, and confirmed sexual activity.

Exclusion criteria were smoking, obesity (BMI > 35), strenuous activity (e.g., frequent heavy lifting), history of previous abdominal/pelvic surgery (aside from vaginal hysterectomy and transvaginal monolateral SF), multiple pregnancies, prolonged labor (>17 hours in nulliparas; >12 hours in multiparas) and fetal macrosomia. Moreover, we also excluded patients with other pelvic disorders such as endometriosis, pelvic inflammatory disease, chronic circulatory, and autoimmune or neoplastic disease, patients who underwent any kind of pharmacological and/or nonpharmacological treatment (including pelvic floor exercises) in the six months preceding the surgery (wash-out period), and patients who were lost after 12 months of follow-up.

### 2.3. Preoperative and Postoperative Evaluation

All of the patients included were treated with TBSF and were evaluated postoperatively at 12 months. To avoid any possible operator-dependent biases, the same surgeon (Diego Rossetti) performed all of the operations.

The preoperative and postoperative physical evaluation was carried out in supine position with the Valsalva test. Prolapse findings were recorded according to the POP-Q landmarks system, as described elsewhere [17]. Women with low anterior compartment prolapse underwent concomitant paravaginal repair. All of the women underwent a preoperative urodynamic evaluation to check for detrusor overactivity or urinary leakage. Women affected by stress urinary incontinence underwent concomitant surgical repair with tension-free vaginal tape-obturator (TVT-O) procedure.

### 2.4. 36-Item Short Form Survey (SF-36)

A SF-36 questionnaire was administered preoperatively and at 12 months, to study the effects of vaginal reconstruction on the quality of life. SF-36 contains 36 questions grouped into 8 categories: physical activity, physical health, physical pain, general health, overall physical vitality, social activity, emotional state, and mental health [18]. The score in each category ranges from 0 to 100 points; the maximum score signifies the highest evaluation of a given category of the quality of life.

### 2.5. Pelvic Organ Prolapse/Urinary Incontinence Sexual Questionnaire-12 (PISQ-12)

A PISQ-12 questionnaire was administered preoperatively and at 12 months; it was used to study the effects of pelvic prolapse on sexual function. PISQ-12 is a short form derived from long-form PISQ-31. It is composed of 12 items and examines three factors: the behavioral emotive factor with items 1–4, the physical factor with items 5–9, and the partner-related factor score with items 10–12. Validity is retained up to two missing values on the short form. If more than two of the 12 values are missing, then the short form no longer predicts long-form scores [19].

### 2.6. Surgical Treatment

In detail, the rectum was caudally and medially pushed by TBSF to avoid injuries. The ischial spines and the sacrospinous ligament were exposed with the help of two long Navratil-Breisky vaginal retractors. According to the standard Richter technique, nonabsorbable monofilament sutures (nonabsorbable 0 suture, coated braided polyester, and double armed with tapercut needle, 48′′) were placed on each side of the sacrospinous ligament for bilateral SF, using a suture capturing device (Capio™ SLIM, Boston Scientific) [14]. A second absorbable 0 suture (absorbable 0 suture, coated braided PGA, double armed with TC tapercut needle, 48′′) was placed bilaterally and medially 1.0 cm from the first suture. The absorbable sutures were attached to the full thickness of the vaginal vault, and the nonabsorbable sutures were placed extramucously. Knots were tied on the fascial side to prevent vaginal erosion due to the nonabsorbable surgical material. To avoid the diversion of the vagina and to achieve minimal tension, the ipsilateral sutures were placed and tagged in the corresponding lateral edge of the vaginal apex. The anterior wall of the vagina was then stitched, using a 2-0 monocryl suture. In all of the cases, patients were not converted to an alternative repair during the operation.

### 2.7. Data Collection

For each patient, we collected data on age, BMI, interval time from monolateral SF to second VPP recurrence, de novo urinary incontinence (after monolateral SF), surgical operating time, blood loss, intraoperative and postoperative complications, pre- and postoperative POP-Q stage, anatomic VPP recurrence after treatment, and preoperative and postoperative results of SF-36 and PISQ-12 questionnaires.

### 2.8. Endpoints of the Study

The primary endpoint in this analysis was to compare each patient's quality of life (through SF-36 questionnaire) and sexual function (through PISQ-12 questionnaire) before and after 12 months from TBSF treatment. The secondary endpoint was to compare the anatomical resolution or decrease of second VPP recurrence after 12 months from TBSF treatment.

### 2.9. Statistical Analysis

#### 2.9.1. Power Analysis

Assuming a standard deviation (SD) of 2 and a mean difference of 1.5 between pre- and postsurgical treatment sexual changes of *p* = 0.05, the sample size calculation indicated that 19 subjects would be the minimum number required for the study to have 90% power.

#### 2.9.2. Statistics Tests

Patients' baseline characteristics were reported as mean ± standard deviation (SD), median (interquartile range), and percentages for continuous and categorical variables, respectively. SF-36 categories and PISQ-12 scores as absolute and difference between preoperative time and postoperative time values were reported as mean ± SD and median (interquartile range). Changes between SF-36 categories and PISQ-12 scores before and after surgical treatment were compared in parametric (paired *t*-test on differences) and nonparametric ways (Wilcoxon signed-rank test on differences), to confirm the reliability of the results. The scores are presented as the mean ± SD. The result was considered statistically significant at *p* < 0.05. All statistical analyses were performed using STATA version 14 (STATA Corp., Texas, USA).

## 3. Results

### 3.1. Patients' Characteristics and Intraoperative and Postoperative Data

Over the study period, we collected data on 23 women affected by symptomatic second recurrence of VPP treated by TBSF that fulfilled our inclusion/exclusion criteria. Three patients were lost at 12-month follow-up (one did not come to the appointment and two refused to answer the questionnaires). Therefore, we presented data analysis of 20 patients.

The mean age for the whole sample was 62.0 ± 5.0 years and mean BMI was 28.3 ± 2.8 Kg/m^2^. The mean interval time from the first SF to second VPP recurrence was 14.6 ± 6.0 months. 20% of the patients (4 women) showed a combination of grade II (Ingelman-Sundberg classification) de novo stress urinary incontinence (SUI) and multicompartment VPP: two of them were affected by Chronic Obstructive Pulmonary Disease (COPD), so probably this element may have played a significant role.

With regard to surgical data, the median operative time was 112 minutes (52–170) and the median estimated blood loss was 150 ml (95–200). No major intra- and postoperative complication occurred (only one patient had postoperative urinary tract infection, successfully treated with oral fluoroquinolones for 5 days). The median hospital stay was 4 days (3–6 days). The general characteristics of the patients and the intraoperative data are reported in Table 1.

### 3.2. Pre- and Postoperative POP-Q Scores Modifications

At 12 months of follow-up we observed a reduction of 4/5 of POP-Q landmarks between pre- and posttreatment. For anterior vaginal wall point Aa and Ba scoring passed from 2.5 ± 2 and 2.5 ± 3 to −2 ± 0.5 and −2.5 ± 0.5, respectively (*p* < 0.001). Considering posterior vaginal wall point, Ap and Bp scoring passed from 1 ± 3 and 2.5 ± 4 to −2.5 ± 0.5, respectively (*p* < 0.001). Point C score significantly improved (*p* < 0.001) between the time before surgery (3.2 ± 1.9) and 12-month follow-up (−2.9 ± 2.1). The total vaginal length (TVL) did not significantly change between pre- and posttreatment. Data are reported in Table 2.

Two patients showed a recurrence of asymptomatic stage II apical prolapse at 12 months.

### 3.3. SF-36 and PISQ-12 Score

With reference to pre- and postoperative quality of life, the results of the SF-36 questionnaire demonstrated a significant improvement for all of the categories (physical activity, physical health, physical pain, general health, overall physical, vitality, social activity, emotional state, mental health, and overall mental) at 12 months after TBSF (*p* < 0.0001 for all domains). In particular, we found the greatest improvements for the “Emotional State” (mean difference 63.77 ± 33.20), “Physical Health” (mean difference 57.61 ± 31.47), “General Health” (mean difference 43.65 ± 15.78), and “Social Activity” (mean difference 37.50 ± 17.27). Complete scores and mean differences for each domain are reported in Table 3.

Concerning pre- and postoperative sexual function, the results of the PISQ-12 questionnaire demonstrated a significant improvement for all three domains (behavioral emotive factor, physical factor, and partner-related factor) at 12 months after TBSF (*p* < 0.0001 for all domains). In particular, we found the greatest improvement for the “Physical Factor” (mean difference 10.83 ± 4.26). Complete scores and mean differences for each domain are reported in Table 4.

## 4. Discussion

In the case of VPP the two most accepted techniques for surgical correction are LSC and vaginal SF [7]. As mentioned before, no randomized trials have been published, investigating the superiority of one of the two approaches [4]. Only data from observational and in particular retrospective series seem to suggest a lower recurrence rate with LSC, though this procedure may be linked to potential intra-abdominal morbidities including sacral hemorrhage, bowel/ureter obstruction, and port site herniation [20]. Furthermore, the estimated learning curve requires almost 60 cases to complete the procedure by laparoscopy without any complications and with a good anatomical outcome in at least 90% of patients [21].

On the contrary, vaginal SF appears to be correlated to lower morbidities [the main complications described are rectal damage (0.4%), pudendal or sciatic nerve injury (1.2%), urinary tract lesions (0.7%), hemorrhage (5.2%), chronic pain (2%), and postoperative urinary tract infections (14.7%)], lower cost, and shorter operating time. Finally, the estimated learning curve for SF required only 20 cases to complete the procedure with a favorable anatomical outcome [5, 6, 22]. However, even if each patient's long-term satisfaction, low morbidity rate, and acceptable cost-effectiveness should be considered the most important variables in the choice of surgery for VPP correction, data regarding these topics are still too scarce to draw any significant conclusion [4, 7]. This is even more remarkable in the case of a second recurrence of VPP. In this circumstance the medical dilemma of whether or not a surgical repair should be performed and what technique should be used is even greater. Adequate guidelines and suggestions are still lacking in the literature [23].

In this study, we assessed the clinical effectiveness of TBFS technique on 20 sexually active postmenopausal patients affected by second VPP recurrence after previous vaginal hysterectomy. At 12-month follow-up, we evaluated both the anatomical outcomes of the surgical procedure and the physical and physiological improvement in the QoL and sexual function. In our opinion, these two fields should always guide clinical practices in patients with pelvic floor dysfunction, but too often they are only considered as secondary outcomes.

Apart from the organic improvement of POP at 12 months, this study clearly demonstrates that TBSF is able to significantly improve both quality of life and sexual function in patients with second recurrence of VVP.

Surgical and technical reasons that could explain our results may be related to the possibility of TBSF in tailoring the individual width of the vaginal apex with bilateral sutures of varying lengths on the sacrospinous ligament. To avoid applying tension on the tissue and decreasing the risk of recurrence, TBSF helped us prevent lateral vaginal deviation along with its disadvantages, keeping its functionality. Moreover, we did not observe any relevant major intra- and postoperative complications. In our study there was no evidence of any particular difficulties in pararectal space dissection and ligament isolation, as described and emphasized in previous published literature [15]. Anatomic recurrence of grade II apical prolapse occurred in two patients (10%) with considerably weak connective tissue on palpation; unfortunately, these patients could not be treated any further because they refused other treatments.

Previous studies have reported anatomic success rates of 77–94% after monolateral SF in smaller cohorts of VVP first recurrence [24–26]. Several authors [27–29] have described 10%–30% anterior support defects after sacrospinous fixation. In addition, because of a slight dorsal-caudal vaginal deviation, traction on the anterior compartment may occur, causing secondary symptomatic grade II cystoceles.

To the best of our knowledge, this was the first study evaluating physical and psychological subjective aspects of quality of life and sexual function (through SF-36 and PISQ-12, resp.) in women affected by second recurrence VVP treated by TBSF. The strengths of our series may be related to the adequate number of patients (although the number is low, it is in line with our power analysis) recruited, the strict inclusion criteria, and the follow-up at 12 months. However, the lack of a control group (including patients undergoing different types of surgical procedures) is an important limitation of our study.

## 5. Conclusions

TBSF appears to be effective and safe for the reduction of POP and for the improvement of quality of life and sexual function in menopausal women affected by second recurrence of VPP, previously treated by monolateral SF. Nevertheless, further randomized trials are needed to compare TBSF with other techniques.

## Figures and Tables

**Table 1:** Clinical characteristics of patients at baseline and intraoperative data.

Variable	% or mean ± SD	Median (IQR)
Age (years)	62.0 ± 5.0	62 (58.0–66.0)
BMI (kg/m2)	28.3 ± 2.8	30 (26.5–29.0)
Parity	1.85 ± 0.8	1 (1-2)
Months to second VVP	14.6 ± 6.0	12.5 (12.0–17.0)
De novo stress urinary incontinence (%)	4 (20.0%)	
Operative time (minutes)	108.3 ± 10.7	112 (52–170)
Blood loss (ml)	139.6 ± 22	150 (95–200)
Hospital stay (days)	3.4 ± 0.6	4 (3–6)

Continuous variables are expressed as mean ± standard deviation (SD) and median (interquartile). Dichotomous variables are expressed as *n* (%). IQR: interquartile range, BMI: body mass index, VVP: vaginal vault prolapse.

**Table 2:** Pre- and postoperative (12-month follow-up) pelvic organ prolapse quantification (POP-Q).

POP-Q variables	Preoperative assessment	Postoperative assessment	*p* value
Aa	2.5 ± 2	−2 ± 0.5	<0.001
Ba	2.5 ± 3	−2.5 ± 0.5	<0.001
Ap	1 ± 3	−2.5 ± 0.5	<0.001
Bp	2.5 ± 4	−2.5 ± 0.5	<0.001
C	3.2 ± 1.9	−2.9 ± 2.1	<0.001
TVL	6 ± 0.5	6 ± 1	NS

Data are expressed as mean ± standard deviation (SD); Aa: anterior vaginal wall, 3 cm proximal to the hymen; Ba: most distal position of the remaining upper anterior vaginal wall; Ap: posterior vaginal wall, 3 cm proximal to the hymen; Bp: most distal position of the remaining upper posterior vaginal wall; TVL: total vaginal length.

**Table 3:** Pre- and posttreatment (12-month follow-up) results of Short Form-36 (SF-36) questionnaires.

Outcome	Pretreatment (T0)		Posttreatment (T0)		Diff (T1 − T0)		*p* value	
	Mean ± SD	Median (IQR)	Mean ± SD	Median (IQR)	Mean ± SD	Median (IQR)	*p* ^*∗*^	*p* ^*∗∗*^
Physical activity	57.8 ± 18.8	50.0 ± 40.0	92.5 ± 9.1	95.0 ± 10.0	34.7 ± 20.4	30.0 ± 40.0	<0.001	<0.001
Physical health	31.5 ± 25.2	25.0 ± 50.0	89.1 ± 16.6	100.0 ± 25.0	57.6 ± 31.5	50.0 ± 75.0	<0.001	<0.001
Physical pain	54.5 ± 16.1	42.0 ± 33.0	87.9 ± 14.5	100.0 ± 26.0	33.4 ± 17.8	26.0 ± 20.0	<0.001	<0.001
General health	25.2 ± 8.8	25.0 ± 10.0	68.8 ± 11.2	72.0 ± 8.0	43.6 ± 15.8	42.0 ± 25.0	<0.001	<0.001
Overall physical	38.6 ± 6.1	39.2 ± 9.2	53.9 ± 4.5	55.4 ± 5.9	15.3 ± 7.5	12.7 ± 11.6	<0.001	<0.001
Vitality	38.7 ± 8.4	40.0 ± 10.0	61.8 ± 7.8	60.0 ± 10.0	23.0 ± 11.3	20.0 ± 15.0	<0.001	<0.001
Social activity	39.1 ± 14.2	37.5 ± 25.0	76.6 ± 10.2	75.0 ± 12.5	37.5 ± 17.2	37.5 ± 25.0	<0.001	<0.001
Emotional state	27.5 ± 25.9	33.3 ± 33.3	91.3 ± 14.9	100.0 ± 33.3	63.7 ± 33.2	66.7 ± 66.7	<0.001	<0.001
Mental health	39.1 ± 8.1	36.0 ± 12.0	67.5 ± 9.6	68.0 ± 12.0	28.3 ± 11.9	32.0 ± 16.0	<0.001	<0.001
Overall mental	31.2 ± 6.1	32.8 ± 9.92	46.9 ± 3.9	47.7 ± 3.2	15.8 ± 7.3	15.1 ± 13.2	<0.001	<0.001

Data are expressed as mean ± standard deviation (SD) or interquartile (IQR). ^*∗*^Paired  *t*-test on differences. ^*∗∗*^Wilcoxon signed-rank test on differences.

**Table 4:** Pre- and posttreatment (12-month follow-up) results of Prolapse/Urinary Incontinence Sexual Questionnaire (PISQ-12) questionnaire.

Outcome	Pretreatment (T0)		Posttreatment (T1)		Diff (T1 − T0)		*p* value	
	Mean ± SD	Median (IQR)	Mean ± SD	Median (IQR)	Mean ± SD	Median (IQR)	*p* ^*∗*^	*p* ^*∗∗*^
Behavioural emotive factor	6.1 ± 2.9	6.0 ± 4.0	10.8 ± 2.6	12.0 ± 2.0	4.6 ± 4.0	5.0 ± 5.0	<0.0001	<0.0001
Physical factor	6.3 ± 2.9	7.0 ± 5.0	17.1 ± 2.4	18.0 ± 3.0	10.8 ± 4.2	11.0 ± 6.0	<0.0001	<0.0001
Partner-related factor	7.0 ± 1.2	7.0 ± 2.0	9.9 ± 1.5	10.0 ± 2.0	2.9 ± 1.5	3.0 ± 2.0	<0.0001	<0.0001

Total score	19.6 ± 5.2	20.0 ± 7.0	37.9 ± 5.3	38.0 ± 8.0	18.3 ± 8.5	20.0 ± 9.0	<0.0001	<0.0001

Data are expressed as mean ± standard deviation (SD) or interquartile (IQR). ^*∗*^Paired  *t*-test on differences. ^*∗∗*^Wilcoxon signed-rank test on differences.
